# Bloodmeal analysis via *COI*-targeted DNA capture and enrichment identifies non-cattle hosts of *Amblyomma variegatum* on St. Croix, US Virgin Islands

**DOI:** 10.1186/s13071-026-07365-6

**Published:** 2026-03-26

**Authors:** Rebecca Ballard, Charles H. D. Williamson, Hayden Kleinschmidt, Greta Buckmeier, Jason W. Sahl, Pia U. Olafson, Jeffery T. Alfred, Joseph D. Busch, David M. Wagner, Denise Bonilla, Nathan E. Stone

**Affiliations:** 1 Pathogen and Microbiome Institute, Northern Arizona University, PO Box 4073, Flagstaff, AZ 86011 USA; 2 USDA, ARS, KBUSLIRL-LAPRU, 2700 Fredericksburg Rd., Kerrville, TX 78028-9184 USA; 3 USDA, APHIS, VS, NVSL, 1920 Dayton Ave, Ames, IA 50010 USA; 4 USDA, APHIS, Veterinary Services, 2150 Centre Ave, Fort Collins, CO 80526 USA

**Keywords:** Tropical bont tick, Bloodmeal analysis, Heartwater disease, African tick-bite fever

## Abstract

**Background:**

*Amblyomma variegatum* threatens the Caribbean cattle industry owing to its role as a vector for *Ehrlichia ruminantium*, the obligate intracellular bacterium that causes heartwater disease, an economically important and potentially fatal ruminant disease. *Amblyomma variegatum* is also a public health concern as a vector for *Rickettsia africae*, the causative agent of African tick-bite fever. Efforts to eradicate *A. variegatum* on Caribbean islands are ongoing to protect cattle from disease and prevent the spread of the vector and diseases to the American mainland. However, reinfestations often occur, possibly owing to the maintenance of ticks by non-cattle hosts that escape treatment. St. Croix in the US Virgin Islands has experienced such eradication challenges.

**Methods:**

To determine whether persistence of *A. variegatum* populations on non-cattle hosts contributes to cattle reinfestation on St. Croix, we analyzed 1 non-attached *A. variegatum* adult female collected from a human and 14 questing adult females collected via cloth dragging along vegetation transects in Lower Love, St. Croix, during 2023. We conducted host bloodmeal analysis by obtaining vertebrate cytochrome c oxidase subunit I (*COI*) sequences from *A. variegatum* DNA extracts using *COI*-targeted DNA capture and enrichment to identify the most recent host bloodmeal. We also screened tick DNA extracts for *E. ruminantium* and *Rickettsia* DNA using *pCS20* Sol1 and PanR8 qPCR, respectively. *Rickettsia* species identification was determined using *ompA* polymerase chain reaction (PCR) and Sanger sequencing, followed by phylogenetic analysis.

**Results:**

We identified vertebrate *COI* sequences for the genera *Capra* and *Canis* in two *A. variegatum* ticks. Although *E. ruminantium* was not detected, DNA from *R. africae* was present in all 15 ticks.

**Conclusions:**

These results suggest that goats, and possibly canines, may serve as alternative hosts for *A. variegatum* on St. Croix, US Virgin Islands, which complicates eradication efforts focused entirely on the treatment of cattle. Fortunately, *E. ruminantium* was not identified in any ticks. However, *R. africae* was ubiquitous, which may be of concern for public health.

**Graphical Abstract:**

**Figure:**
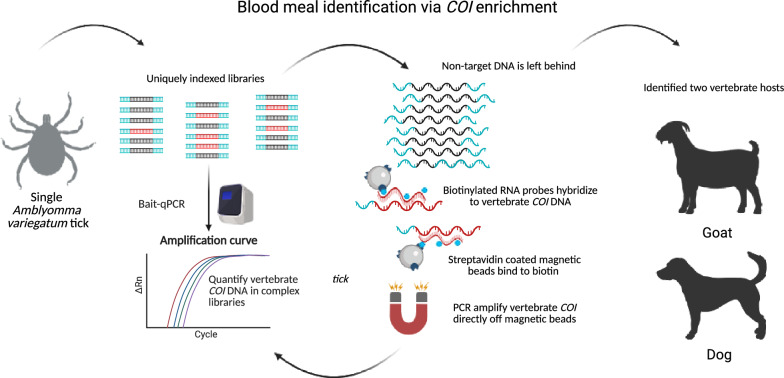

**Supplementary Information:**

The online version contains supplementary material available at 10.1186/s13071-026-07365-6.

## Background

The tropical bont tick (*Amblyomma variegatum*) transmits the cattle pathogen *Ehrlichia ruminantium*, an obligate intracellular bacterium and causative agent of heartwater disease, which is associated with high mortality and economic losses in ruminants [1]. *Amblyomma variegatum* infestations on cattle also are linked to increased dermatophilosis caused by the actinomycete *Dermatophilus congolensis*, resulting in hide damage, livestock deaths, and substantial economic losses [1–3]. Ongoing efforts to eradicate *A. variegatum* on Caribbean islands are conducted primarily to protect cattle from heartwater disease and prevent the spread of diseases and the vector to the American mainland. Despite numerous eradication efforts since its introduction from Africa in the early 1800s [4], several Caribbean islands remain infested [5]. The Caribbean *Amblyomma* Program (CAP) was initiated to address this challenge, but reinfestation of treated pastures is often experienced within a few years of eradication [6]. *Amblyomma variegatum* ticks primarily infest domestic animals, such as cattle, goats, sheep, and dogs [4] but are also known to infest migratory cattle egrets (*Ardea ibis*) [2, 7]. The use of non-cattle hosts, especially goats [8], could allow tick populations to persist and reinfest livestock after eradication on cattle, but the role of such hosts in the maintenance of *A. variegatum* on St. Croix remains unclear.

Because attempts to eradicate *A. variegatum* on St. Croix, US Virgin Islands (USVI), have remained challenging [6], we investigated the possible role of non-cattle hosts in the persistence of *A. variegatum* on this island using host bloodmeal identification with a novel *COI*-targeted DNA capture and enrichment approach. We also made a direct comparison with the traditional approach to bloodmeal identification using polymerase chain reaction (PCR) and sequencing of the mitochondrial cytochrome c oxidase subunit I (*COI*) gene as previously described [9]. In addition to its role as a vector for heartwater disease, *A. variegatum* poses a public health risk as a vector for *Rickettsia africae* (African tick-bite fever) [7], which is widespread in the Caribbean islands, affects local residents, and may impact travelers to the region [10–12]. As such, we screened all 15 ticks for both *E. ruminantium* [13] and *Rickettsia* species [14] DNA via quantitative PCR (qPCR).

## Methods

### Tick collection and DNA extraction

As part of a surveillance program for tropical bont ticks conducted on a premise known to have tick-infested animals, 14 adult female ticks were collected in May 2023 by environmental convenience sampling using the cloth-dragging method and carbon dioxide (CO_2_) traps; 1 other was collected from a human (unattached tick). Dragging was opportunistic, with a 1 m^2^ drag cloth focusing on areas of ecotone and animal resting areas. The CO_2_ trap was assembled and monitored during this time so that 30 min of joint dragging and CO_2_ trapping were performed. Sampling occurred early in the day near sunrise when wind and temperature were low. The site is pasture for cattle, sheep, goats, and horses, and occasionally has dogs to herd livestock. It adjoins another infested pasture with a low single wire fence. The predominate vegetation class is grassland with small trees and interspersed shrubbery within (Additional File 1: Supplementary Fig. S1). The ticks were removed from the CO_2_ trap or drag cloth, preserved in 70% ethanol, and shipped to the US Department of Agriculture–Animal and Plant Health Inspection Service (USDA–APHIS), National Veterinary Services Laboratories (Ames, IA, USA) for identification using taxonomic keys [15]. For DNA extraction, ticks were removed from ethanol and immersed in 1% sodium hypochlorite for 30 s, rinsed three times in sterile water, and ground with a liquid nitrogen-cooled pestle. DNA was then extracted from individual adult ticks using the DNeasy Blood and Tissue Kit (Qiagen, Valencia, CA, USA), and DNA was eluted in a 100 µl volume. Sample details are provided in Additional File 2: Supplementary Table S1.

### Host bloodmeal analysis

#### Nested PCR protocol

The universal host bloodmeal identification protocol described by Alcaide and colleagues [9] was attempted utilizing the published conditions, with forward primer BC-FW and reverse primer BCV-RV1 in the first PCR reaction and BC-FW and reverse primer BCV-RV2 in the second. However, instead of an M13-tail for reamplification, we attached universal tails UT1 5′ACCCAACTGAATGGAGC to the 5′ end of BC-FW and UT2 5′ACGCACTTGACTTGTCTTC to the 5′ end of BCV-R2 to make the amplicons compatible with our Amplicon sequencing (AmpSeq) workflow [16–18].

#### DNA capture and enrichment

We hypothesized that we would improve the sensitivity of bloodmeal identification by employing the Agilent SureSelect DNA capture and enrichment system (Agilent Technologies, Santa Clara, CA, USA), which relies on 120-base pair (bp) RNA probes to “capture” DNA from specific targets. We designed probes targeting vertebrates by parsing *COI* sequences from the BOLD database [19] that were annotated as phylum “Chordata”; this analysis returned a total of 169,122 sequences. Sequences were clustered with USEARCH version 11.0 [20] at 90% identity, resulting in a total of 17,192 sequences, which were then sliced into 120-nucleotide (nt) fragments, overlapping by 60 nts. These candidate probes were clustered with USEARCH at an identity of 90%, resulting in 141,010 candidate probes. To remove probes that could hybridize to arthropod sequences, probes were aligned against the *COI* from *Ixodes scapularis* (as a representative tick species) with LS-BSR version 1.3 [21] in conjunction with BLAT version 35 [22]; and any probe with a blast score ratio (BSR) [23] value > 0.6 was removed (*n* = 112). This analysis was also performed for *Aedes* species (as a representative mosquito species), which removed an additional 297 potential probes. Finally, the probes were aligned against *COI* sequences from several non-chordates, and any probes that aligned were removed. The final probe set, consisting of 139,091 probes, was aligned against *COI* sequences from several diverse hosts (humans, birds, deer, mice, tortoises) with minimap2 version 2.26 [24] to ensure full coverage.

Illumina-compatible libraries were prepared according to the Agilent SureSelectXT protocol, and as previously described [25], with the following modifications: To accommodate dual indexing, a second index adaptor was ligated to the ends of the DNA fragments using the SureSelectXT Ligation Master Mix, followed by bead purification. Each ligated fragment was uniquely indexed through PCR amplification following the manufacturer’s protocol for 14 cycles (2 min at 98 °C, 14 cycles for 30 s at 98 °C, 30 s at 60 °C, 1 min at 72 °C, and a final extension of 5 min at 72 °C), followed by a second bead purification.

A slow-hybridization protocol was employed following the manufacturer’s recommendations and as previously described [26]. Approximately 1500 ng of the total library was hybridized at 65 °C for 16–24 h with RNA probes. The increase in *COI* signal from DNA recovered after enrichment was quantified by qPCR using primer pair *ln1*f, 5′GGNGAYGAYCARATNTACAATGT and *ln1*r, 5′GGNGGNAGNAGTCARAARC [27]; cycle threshold (Ct) values are reported in Additional File 2: Supplementary Table S1. On the basis of this outcome, a second round of DNA capture and enrichment was conducted for 4 of the 15 samples. The qPCR assay was performed on the sequence-ready libraries prior to enrichment using ~20 ng of input DNA and then after each round of enrichment using ~1 ng of input DNA. PCRs were carried out in 10 µL volumes and in duplicate reactions containing the following reagents: 1 µL of diluted DNA template, 1× SYBR^®^ Green Universal master mix (Applied Biosystems, Foster City, CA, USA), and 0.2 µM of each primer. The assay was run on an Applied Biosystems 7500 Fast Real-Time PCR System with SDS 7500 software version 2.0.6 under the following conditions: 95 °C for 10 min, and 40 cycles of 95 °C for 15 s and 58 °C for 1 min; human and mouse DNA were included on each run as positive controls, and non-template controls were also included. Enriched libraries were assessed for quality and quantity, and were pooled in equimolar amounts prior to sequencing, as previously described [26]. Sequencing was performed on an Illumina MiSeq Instrument with a v3 600 cycle (2 × 300) kit (Illumina, San Diego, CA, USA).

Paired-end Illumina reads were classified with Kraken 2 version 2.1.3 [28]. Unique *COI* sequences were downloaded from the MIDORI2 database version GB264 [29]. Sequences with taxonomic classifications of “Chordata” and “Ixodida” were selected, and a custom Kraken 2 database was created by adding this set of sequences to the Kraken 2 Univec_Core database. Adapters were trimmed from paired-end Illumina reads with cutadapt version 4.9 [30] (-m 100 -q30 -a AGATCGGAAGAGCACACGTCTGAACTCCAGTCA -A AGATCGGAAGAGCGTCGTGTAGGGAAAGAGTGT), and trimmed reads were classified with Kraken 2 at a confidence score threshold of 0.3. Kraken 2 output reports for multiple samples were combined with KrakenTools version 1.21 [31]. Taxonomic classifications were analyzed at the genus level, and only genera with > 100 assigned reads for a sample were considered. To confirm and further evaluate taxonomic classifications, paired-end Illumina reads for samples 1209 and 1215.1 were aligned to *COI* sequences representing the genera *Capra* (AB736087.1) and *Canis* (OQ341081.1:5350–6894) with minimap2 version 2.28 (-ax sr) [24]. Depth of coverage at each position of the reference sequence was calculated with samtools version 1.21 [32], and consensus sequences were constructed with the samtools consensus utility (positions with less than 10× coverage were not considered for the consensus sequences). Consensus sequences were searched against the core_nt database [32, 33] with BLASTN version 2.12.0 [34, 35]. Consensus FASTA files are provided in the Supplementary Materials and GenBank (see data availability statement below).

### Pathogen detection

*Ehrlichia ruminantium*-specific qPCR was performed using the *pCS20* gene Sol1^SG^ assay [13], and ~20 ng of *A. variegatum* DNA was screened in duplicate 10 μl reactions containing 1× SYBR^®^ Green Universal Master Mix and 0.25 µM of each primer. The assay was run on an Applied Biosystems 7500 Fast Instrument under the following conditions: 95 °C for 10 min and 40 cycles of 95 °C for 15 s and 51 °C for 1 min, followed by amplicon dissociation. A 134-bp gBlock (IDT, Coralville, IA, USA) was designed to flank the Sol1^SG^ priming sites (underlined and lowercase in Sol1_gBlock, 5′GCACCAAGTTATCATATAATAAacaaatctggtccagatcacAATCCTTGTTTTACTGTAGAAGTAAGGATCAATTCACATGAAACATTACATGCAACTGGTCATAACAAAAAactagctgaacagaaagctgC) and used as a positive control for each PCR at a concentration of 1 × 10^–6^ ng/µL; non-template controls were also included. A dissociation temperature of 74.2 ± 0.5 °C is expected for *E. ruminantium* amplicons [13].

*Rickettsia* species qPCR was performed using the PanR8 TaqMan PCR primers and probes [14] in duplicate 10 μL reactions containing ~20 ng of tick DNA as template and the following reagents: 1× PCR buffer, 1× ROX dye, 5 mM MgCl2, 0.2 mM dNTPs, 0.02 U/µL Platinum^®^ Taq polymerase, 0.5 µM of each primer (PanR8_F and PanR8_R), and 0.25 µM of the PanR8_P probe. We utilized the following conditions on an Applied Biosystems 7500 Fast instrument: 95 °C for 8 min to release the polymerase antibody, followed by 45 cycles of 95 °C for 5 s and 60 °C for 30 s. Ct values < 35 were considered positive. Genomic DNA from an isolate of *R. rhipicephali* was used as a positive control; non-template controls (sterile water) were also included. Spotted fever group *Rickettsia* species identification was accomplished for PanR8-positive samples by Sanger sequencing of a ~632-bp region of the *OmpA* gene. The “*OmpA* primary” PCR primers [14, 36] were used for amplification and sequencing with previously described conditions [37]. Sequence chromatograms were edited manually in SeqMan Pro version 17 (DNASTAR, Madison, WI, USA), and primers were trimmed resulting in a 590-bp fragment. Paired consensus sequences were identified by comparison with the public database using National Center for Biotechnology Information (NCBI) BLASTN and by phylogenetic analysis against a reference set of spotted fever group *Rickettsia* spp. A maximum likelihood phylogeny was inferred on a 590-bp *OmpA* alignment using IQ-TREE version 2.2.0.3 with default parameters, 1000 bootstraps replicates [38], and the integrated ModelFinder method [39]. The phylogeny was rooted with *Rickettsia amblyommatis* strain Darkwater (GCA_000964995.1).

## Results

The traditional *COI* nested PCR protocol did not yield any detectable target amplicon in these samples after the first or second PCRs, likely owing to extremely low quantities of host DNA present in these *A. variegatum* ticks and/or DNA digestion in the midgut. However, our DNA capture and enrichment protocol yielded vertebrate and invertebrate *COI* reads. At the family level, reads were assigned to Ixodidae for all samples, and two potential bloodmeal taxa were identified—Bovidae (samples 1209 and 1212.2) and Canidae (samples 1209 and 1215.1) (Additional File 3: Supplementary Table S2). At the genus level, *Amblyomma* was identified in every sample, as expected. For most samples, this was the only genus-level classification identified (at > 100 classified reads). For sample 1209, a subset of reads was classified as both *Capra* and *Canis* and, for sample 1215.1, a subset of reads was classified as *Canis* (Table 1). For sample 834, some reads were classified as *Rhipicephalus*, which could be low-level contamination from sample handling or laboratory processing or misassignment in Kraken 2.

**Table 1:** Read counts of mitochondrial cytochrome c oxidase subunit I assigned to the genus level in DNA extracts from 15 *Amblyomma variegatum* from St. Croix, US Virgin Islands

Family	Genus	1209	1210.1	1210.2	1210.3	1210.4	1210.5	1211	1212.1	1212.2	1214	1215.1	1215.2	759.1	759.2	834
Ixodidae	*Amblyomma*	72190	39385	42427	5634	20312	1158	41625	4825	34110	54143	194413	12120	87798	15965	86245
Ixodidae	*Rhipicephalus*	0	0	4	0	0	0	0	0	0	0	0	0	0	0	112
Bovidae	*Capra*	**2749**	0	0	0	0	0	0	0	1	0	0	0	0	0	0
Canidae	*Canis*	**383**	0	0	0	0	0	0	2	0	0	**1297**	0	0	0	0

Only taxa with at least 100 classified reads at the genus level for at least one sample are presented. Bold font indicates >100 classified reads for a vertebrate taxon.

BLAST results for consensus sequences support the *Capra* and *Canis* taxonomic classifications, displaying 100% sequence identity to *Capra hircus* (domestic goat) and *Canis lupus familiaris* (dog), respectively. To further evaluate vertebrate bloodmeal taxonomic classifications, paired-end Illumina reads for samples 1209 and 1215.1 were aligned to *COI* sequences representing the genera *Capra* (AB736087.1) and *Canis* (OQ341081.1:5350–6894). For sample 1209, reads mapped along the entire 1545-bp length of the *Capra* reference sequence (Additional File 4: Supplementary Fig. S2A). In the *Canis* reference, reads mapped to shorter regions of the reference sequence—tick 1209 had a 166-bp region at the beginning of the reference, whereas tick 1215.1 had a 195-bp region closer to the 3′ end of the reference (Additional File 4: Supplementary Fig. S2B).

**Fig. 1:**
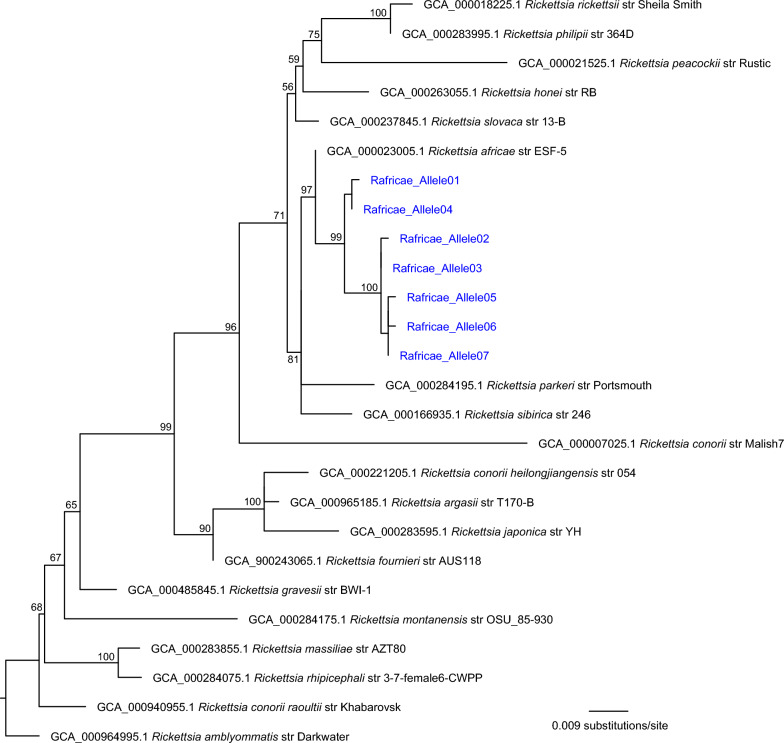
Maximum likelihood phylogeny inferred from a 590-nucleotide partial alignment of the *OmpA* gene and rooted with *Rickettsia amblyommatis* strain Darkwater (accession no. GCA_000964995.1). Bootstrap values are indicated on branch nodes. Blue text indicates sequences that were generated during this study, whereas black sequences were included as references and downloaded from GenBank; accession numbers are listed in the annotations for each sequence. Genus and species designations are also included in the annotations

*Ehrlichia ruminantium* was not detected in any of the 15 tick samples, but *Rickettsia* was detected in all 15 ticks via PanR8 qPCR, with Ct values ranging from 22.66–25.19 (Additional File 2: Supplementary Table S1). Sanger sequencing of the *OmpA* gene for all 15 ticks revealed seven unique *Rickettsia* sequences among them, sharing 98.81–99.66% identity to *R. africae* (Additional File 2: Supplementary Table S1) and grouping in a phylogenetic clade with *Rickettsia africae* strain ESF-3 (Fig. 1); each tick also carried two alleles, suggesting a gene duplication in the *R. africae* they harbored (Additional File 2: Supplementary Table S1). Representative sequences for all seven alleles are available in Additional File 5: Supplementary Table S3 and GenBank.

## Discussion

Our host bloodmeal analysis suggests that *A. variegatum* may be feeding on non-cattle hosts on St. Croix, such as goats and free-roaming dogs, which could undermine eradication efforts that focus only on treatment of cattle. *Amblyomma variegatum* ticks have been widely observed to infest goats across Africa [40] and are reported on dogs [41, 42], including on various Caribbean islands [43]. Although *A. variegatum* has persisted in the Caribbean for over a century, its maintenance beyond cattle infestations has remained largely unexplained. At our USVI field sites, infested premises have a mixture of sheep, goats, cattle, and horses with livestock dogs onsite. Additionally, white-tailed deer (*Odocoileus virginianus*), feral goats, and birds such as cattle egrets roam freely elsewhere throughout the island, complicating the landscape ecology. Fencing is often poor and knowing the ownership of livestock is sometimes complicated by goats, sheep, and horses that roam or are tethered outside of fenced premises. By applying a novel *COI*-targeted DNA capture and enrichment technique, this study provides molecular evidence that *Capra* and *Canis* species are possible non-cattle hosts for *A. variegatum* on St. Croix, insights that could inform more effective surveillance and eradication strategies.

*Rickettsia africae* appears to be common in *A. variegatum* ticks collected on St. Croix, potentially representing a significant public health threat because African tick-bite fever is a neglected emerging infectious disease that affects people living or visiting endemic areas [10]. To illustrate this threat, one of the *Rickettsia*-positive ticks was collected unattached from a human. Further, the detection of *R. africae* DNA in all 15 ticks highlights the potential of non-bovine pathogen reservoirs for *R. africae*, as 14/15 ticks were collected via vegetation dragging, rather than directly from cattle. These ticks could possibly have been infected with *R. africae* via horizontal transmission [44] by feeding on a non-cattle host reservoir, a hypothesis that is supported by other studies that identified *R. africae* antibodies in goats and sheep across the Caribbean islands [10]. In addition, *R. africae* can also be transmitted vertically [45, 46], and this would contribute to a high positivity rate in a local tick population.

## Conclusions

Targeted DNA capture and enrichment of *COI* sequences offers an exciting new approach for identifying host sources of vector bloodmeals. In this study, this approach successfully identified host bloodmeals in two tick samples that failed using a traditional nested PCR approach. This could be because the nested PCR approach targets a specific region of the *COI* gene that may have already been digested in the tick’s midgut, whereas hybridization probes can capture even highly fragmented *COI* sequences in a non-targeted manner. Thus, DNA capture and enrichment may provide an increased window of opportunity for successful identification of arthropod vector host bloodmeals. For the 13 samples where bloodmeal identification was not determined with either approach, it is possible that the ticks had either not consumed a bloodmeal prior to capture [47] or the most recent bloodmeal was completely digested. Although our DNA capture and enrichment probes were designed to hybridize to vertebrate *COI* sequences and exclude invertebrate sequences, many invertebrate *A. variegatum* *COI* sequences were still present (and sequenced) in the final enriched libraries. This was owing to nucleotide similarities among vertebrate and invertebrate *COI* sequences and the overpowering abundance of *A. variegatum* *COI* sequences relative to the vertebrate host bloodmeal *COI* sequences present in pre-enriched samples that were retained after enrichment. Regardless, our *COI* capture and enrichment system provided robust amplification of host bloodmeal *COI* sequences from two ticks and facilitated insights into possible non-cattle hosts of *A. variegatum* on St. Croix to better inform eradication efforts.

## Supplementary Information

Supplementary Material 1. Figure S1. Photo of sampling site at Lower Love, St. Croix, 2023. A pressurized CO_2_ canister (visible in the foreground) supplies two gas lines going opposite directions, one line for each tick trap. One of the white cloth traps is visible between the gate and a small woodpile. Cattle at this site can be seen in the middle of the photo.

Supplementary Material 2. Figure S2. Depth of coverage of reads for two samples aligned to reference *COI *sequences.

Supplementary Material 3. Table S1. Sample metadata with GenBank accession numbers, qPCR results and pathogen detection. Table S2. Species level *COI *read counts in 15 *Amblyomma* *variegatum* from St. Croix. Table S3. *Rickettsia africae* fasta consensus sequences and GenBank accession numbers for seven alleles identified within 15 *Amblyomma* *variegatum* from St. Croix. Table S4. Consensus sequences of *Capra* and *Cani*s *COI* reads from two samples.

## Data Availability

The data supporting the conclusions of this article are included within the article and its additional files. Sequences are deposited in GenBank under BioProject PRJNA1314289 and individual accession numbers are provided in the supplementary materials (Additional File 5: Supplementary Table S3). Consensus sequences of Capra and Canis *COI *reads are available in Additional File 6: Supplementary Table S4.
