# Supplementary figures and images for: Bloodmeal analysis via COI-targeted DNA capture and enrichment identifies non-cattle hosts of Amblyomma variegatum on St. Croix, US Virgin Islands
Source: Parasit Vectors. 2026 Mar 26;19:199. doi: 10.1186/s13071-026-07365-6 (PMC13141529; doi:10.1186/s13071-026-07365-6)

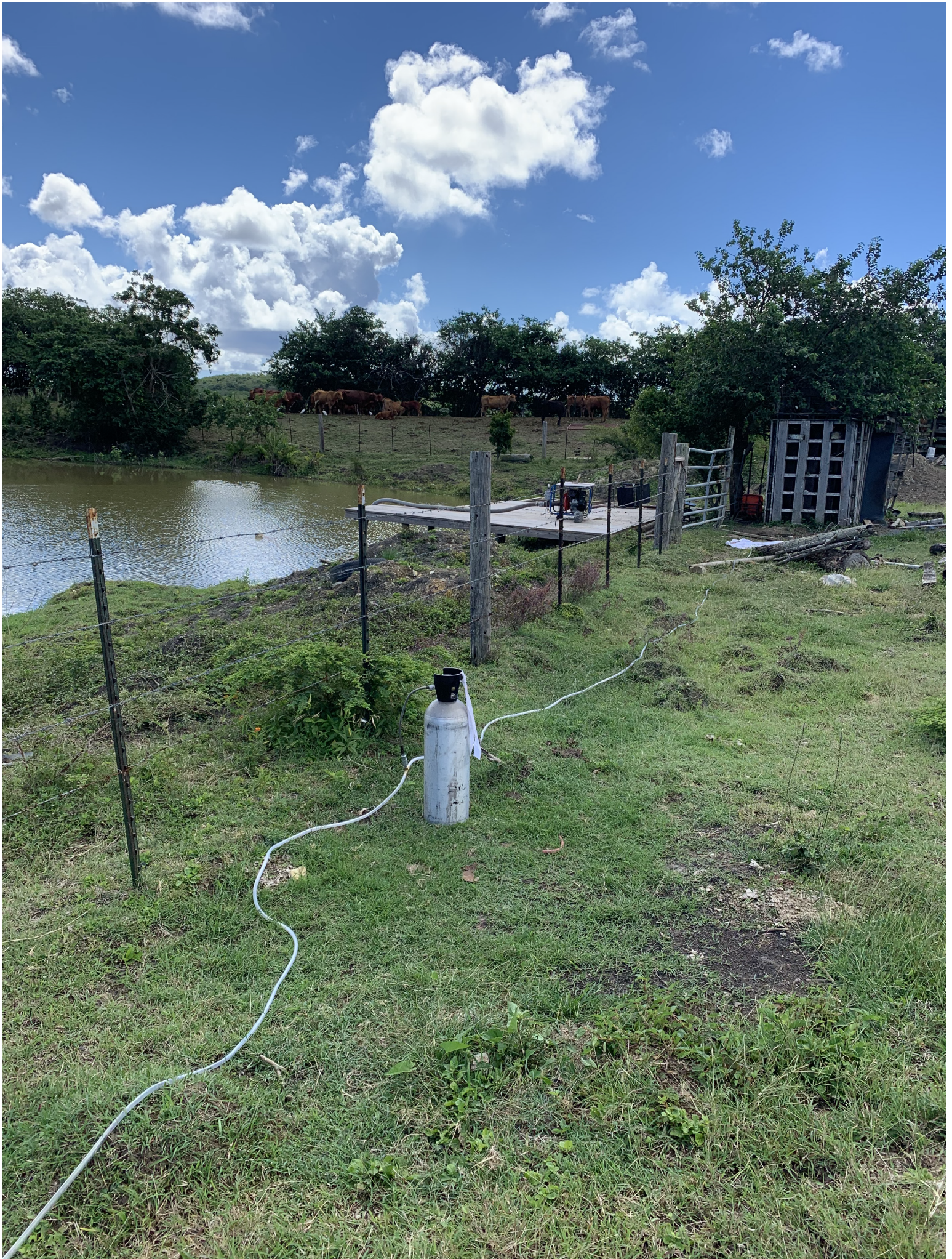

Supplement: Supplementary file 1 — Supplementary Material 1. Figure S1. Photo of sampling site at Lower Love, St. Croix, 2023. A pressurized CO2 canister (visible in the foreground) supplies two gas lines going opposite directions, one line for each tick trap. One of the white cloth traps is visible between the gate and a small woodpile. Cattle at this site can be seen in the middle of the photo. [file 13071_2026_7365_MOESM1_ESM.pdf]

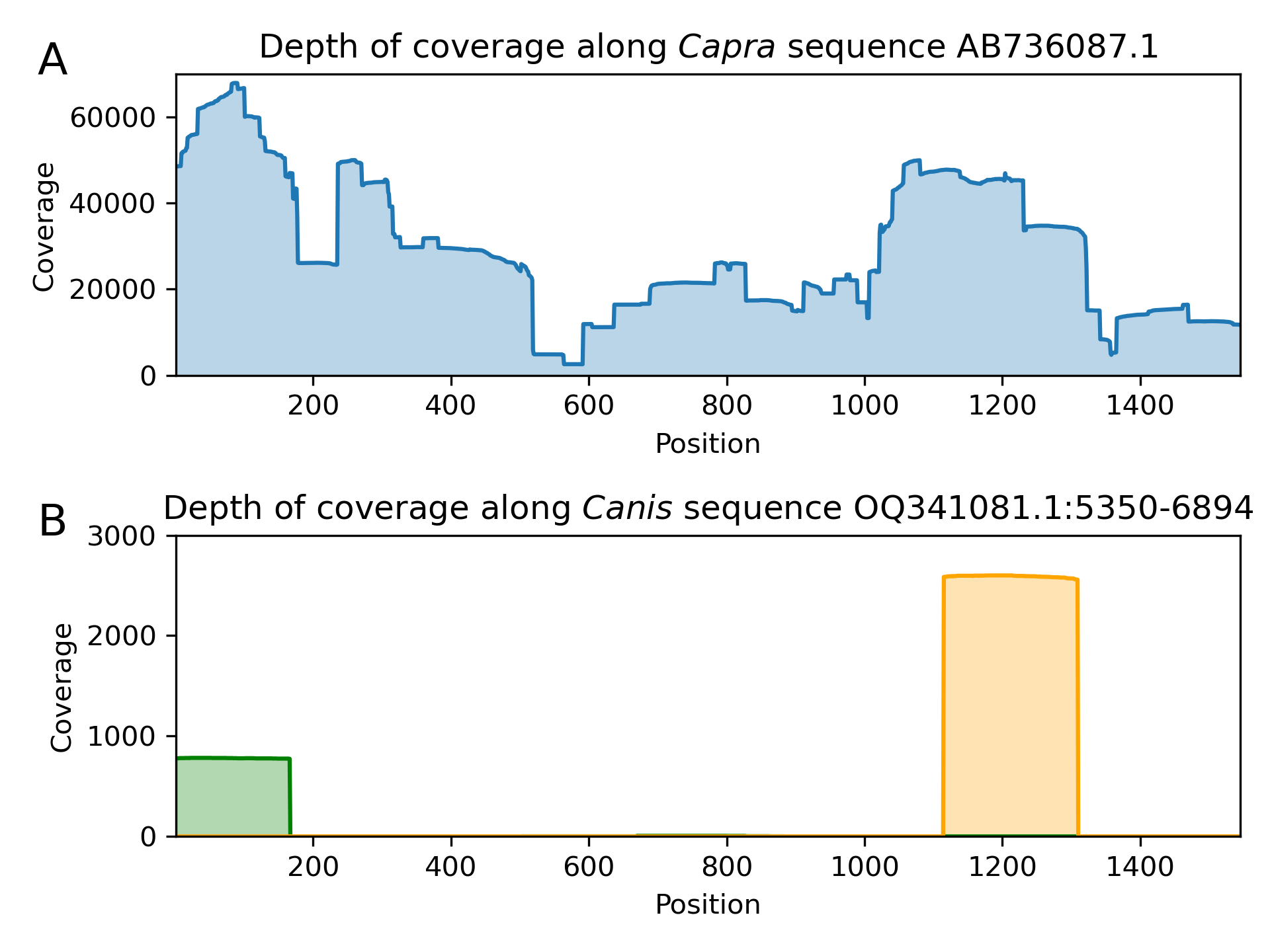

Supplement: Supplementary file 2 — Supplementary Material 2. Figure S2. Depth of coverage of reads for two samples aligned to reference COI sequences. [file 13071_2026_7365_MOESM2_ESM.tiff]
